# Assessing children’s potential exposures to harmful metals in tire crumb rubber by accelerated photodegradation weathering

**DOI:** 10.1038/s41598-023-38574-z

**Published:** 2023-08-24

**Authors:** Robyn Winz, Lee L. Yu, Li-Piin Sung, YuYe J. Tong, Dejun Chen

**Affiliations:** 1 Environmental Metrology and Policy Program, Graduate School of Arts and Sciences, Georgetown University, Washington, DC 20057 USA; 2 National Institute of Standards and Technology, Gaithersburg, MD 20899 USA

**Keywords:** Environmental chemistry, Environmental monitoring

## Abstract

Whether a tire crumb rubber (TCR) playground would expose children to potentially harmful chemicals such as heavy metals is an open question. The released metals available for pickup on the surface of TCR tiles was studied by accelerated 2-year aging of the TCRs in the NIST-SPHERE (National Institute of Standards and Technology Simulated Photodegradation via High Energy Radiant Exposure). The dermal contact was mimicked by a method of composite surface wiping from US Environmental Protection Agency throughout the weathering process. The surface release of ten most concerned harmful metals (Be, Cr, Cu, As, Se, Cd, Sb, Ba, Tl, Pb) was monitored through the course of aging. The cumulative release of Cu, As, Tl, and Sb reached potentially harmful levels at various times within 3 years, although only Cr was found at a harmful level on the surface of the tiles. Taking the cleansing effect of precipitation or periodic cleansing with rain into account, TCR playgrounds may still be safe for use.

## Introduction

Recycling or repurposing end-of-life tires is an economic practice for recovering energy and materials. According to the U.S. Tire Manufacturers Association (https://www.ustires.org/scrap-tire-markets), end-use markets consumed 81.4% of scrap tires generated in the U.S. in 2017, which includes tire-derived fuel (> 43%), tire crumb rubber (TCR) applications (≈ 25%), civil engineering products (≈ 7.9%), and other markets (≈ 7.4%), and accounts for more than 205 million scrap tires. About 25% and 22% of the TCR were used in sports surfacing and playground tiles/mulches, respectively, and their use is expected to increase further because TCR can absorb shock and reduce physical injuries^1,2^. Yet there have been increasing concerns over the potential human exposure to harmful chemicals including organic contaminants and metals after repetitive physical contact with the aging TCR products, particularly on children’s playgrounds^2–4^.

The U.S. Consumer Product Safety Commission completed a survey of American households on child interaction and potential exposure to playground surfacing materials in 2019. It reported that more than half of children visited playgrounds at least once a week with each visit lasting 30–59 min, and more than one-third spent 60–120 min at playgrounds per visit^5^. Such high-frequency visits would likely expose children to known and unknown harmful chemicals via dermal (for instance by hands) contact, ingestion, and/or inhalation that could cause adverse health effects.

The released substances that previous studies have identified from consumer products made of TCR granules include polycyclic aromatic hydrocarbons^6–13^, phthalates^10,12,13^, vulcanization additives^11,13^, and metal elements such as Al, As, Ba, Ca, Co, Cr, Cu, Cd, Fe, K, Li, Mg, Mo, Ni, Pb, Se, Sr, Tl, V, and Zn^7–12,14–18^. The organic contaminants probably resulted from the decomposition of rubber polymers and vulcanization accelerators, plasticizers during tire shredding and grinding; the metals primarily originated from nature rubber itself, the metal oxides catalysts for vulcanization and residuals of steel belt wires for the tire shreds and chips^1^. In comparison to organic contaminants, the metals are nondegradable and persist in the recipient environments, thus the long-term accumulation of toxic metals is of particular concern. Secondly, the metals were found to continue to leach out over the 30-day experimental period but concentrations of organic chemicals in the leachates stabilized within days^19^. However, the mechanism of TCR aging with sunlight, heat, moisture/rain, oxygen/ozone are poorly understood. Few studies found that the oxygen/ozone, ultraviolet (UV) radiation and heat could accelerate the oxidative degradation of vulcanizates or destroy the antidegradants on the surface; and that water from moisture/rain could cause the leaching of soluble components^20–23^.

Strong acid extraction techniques have been developed to assess the bulk metal contents in TCR products^9,24,25^. For example, Cd ranging from 0.09 to 1.39 mg/kg and levels of Pb from 1.9 to 33.1 mg/kg were found in commercially available TCR mulch after strong acid digestion with microwave^25^. Twenty four metals were identified in 13 artificial-turf playfields; among these, Zn (1–19 g/kg) exceeded substantially the pertinent standards, by up to two orders of magnitude^9^. Furthermore, weak acid or outdoor solutions such as seawater have been applied to simulate the effect of real-life weathering on the slow leaching of metals^13,19,26^. An Italian study found that the most abundant metals were Zn, Fe, and Co in seawater leachate^19^. Testing directly the drainage from artificial turf fields was also employed by carefully controlled experiments in which the concentrations of heavy metals, except for Zn (up to 0.5 mg/L), were found to be low^16–18,27,28^.

Bear in mind that only the metals on the surface of TCR can be taken up through direct human contact; therefore, the amount of metal available on the surface should be used to assess potential human exposure. The metal contents of digests, leachates, or drainage are TCR’s bulk properties, which are far from satisfactory in estimating human exposure because the pathways of human exposure to metals below the surface are largely unknown. A more useful approach for evaluating potential human exposures is to assess the weathering-dependent metal surface release (MSR) from TCR materials, particularly those having long environmental durability, such as children’s playground tiles. Such studies have been rarely found in the literature thus far, and relevant data are therefore very limited if ever existed^29^.

To help address this critical knowledge gap, herein, an accelerated weathering-dependent study of the MSR from commercially available TCR playground tiles of ten selected metals (Be, Cr, Cu, As, Se, Cd, Sb, Ba, Tl, Pb) chosen for their regulation under the Safe Drinking Water Act was reported^30^. The MSR was captured by the US EPA (Environmental Protection Agency) composite wiping method used for assessing the MSR of Pb in residential areas^31^. The accelerated weathering of the TCR playground tiles under heavy UV irradiation (12.1 MJ/m^2^/day), relative humidity of 0% (dry) and 75% (wet) with a constant temperature of 55 °C in air was achieved by using NIST’s (National Institute of Standards and Technology) SPHERE (Simulated Photodegradation via High Energy Radiant Exposure)^32–37^, which has been successfully applied to weathering studies of polymers^33,34^ and nanocomposites^35,36^. Notice that the oxidative degradation by ozone, removal of metal ions by rain, removal of degraded elastomer by the mechanical effect of rain which can expose inner layer of polymer matrix are not simulated with SPHERE. However, using the EPA composite wiping method could remove the degraded elastomer matrix on the surface and expose the new material from initially below the surface.

The amount of metal from MSR was determined by ICP-MS (inductively coupled plasma mass spectrometry). For the ten selected metals in this study, six weathering-dependent MSR patterns were observed over a span equivalent to 2 years. The cumulative MSR allowed us to determine if an element of interest would reach a harmful level on the surface over a certain period of weathering. Since Pb is the only element that has a regulatory limit for its surface contents, the potentially harmful surface content levels for other elements were estimated from regulatory limits for their bulk content using a surface-to-bulk content ratio (StB, vide infra) of 0.025 derived from EPA regulations for Pb on the surface and in the bulk.

## Methods and materials

### Selection of samples

Six samples of commercially available TCR playground tiles were provided voluntarily by two manufacturers, A and B. The two major hardware stores carry several of Manufacturer A’s products. Manufacturer B is relatively smaller with over 200 million square feet of rubber flooring delivered each year. The specification of Samples S1 through S6 is as follows:

**Table:** 

Sample #	Materials	Surface color	Manufacturer
S1	60% post-consumer recycled rubber	Black	A-product 1
S2	60% post-consumer recycled rubber	Green	A-product 1
S3	Recycled SBR rubber	Black	B-product 1
S4	Recycled SBR rubber	Blue	B-product 1
S5	60% post-consumer recycled rubber	Black	A-product 2
S6	60% Post-consumer recycled rubber	Black	A-product 3

*SBR: Styrene-Butadiene Rubber.

### SemiQuant measurements of metals in the samples

Three replicates of each in the above sample list were prepared for the SemiQuant survey measurement by a model 7500cs ICP-MS (Agilent, Santa Clara, CA, USA). Prior to microwave digestion of the TCR tiles, each replicate was transferred to a cleaned model MM400 ball mill (Retsch, Haan, Germany) and a sufficient amount of liquid nitrogen was added to chill the ball mill and render the sample brittle for cryo-milling. Each replicate was homogenized at 15.0 Hz for 4 min and transferred into a clean vessel. About 0.25 g of the sample was weighed on a balance (m_1_), and transferred into a Teflon EasyPrep microwave vessel for digestion in a Mars 6 microwave (CEM, Matthews, NC, USA). Approximately 10 mL of Optima Grade (Fisher Scientific, Fair Lawn, NJ, USA) nitric acid was added into the vessel and the sample was digested at the pressure of 800 psi and temperature of 220 °C for 15 min. The temperature ramp speed was 25 °C/min from room temperature to 220 °C. The replicates from Samples S1 to S3 were fully digested but those from Samples S4 to S6 contained white precipitates in powder form which were centrifuged out before ICP-MS analysis. All digested replicates were diluted to about 50 g in pre-weighed 60 mL low-density polyethylene (LDPE) bottles (m_2_) with water. The total weight of the replicate and bottle (m_3_) was recorded.

A 1–2 mL aliquot of each digest was transferred into a pre-weighed 15 mL Falcon tube (m_4_) and the total mass was weighed and recorded (m_5_). A 1–2 mL aliquot of diluted rhodium as an internal standard (26.55 ng/g from NIST SRM 3144, lot 170930) was added into the tube and weighed (m_6_). The final solution was diluted to about 10 mL and weighed (m_7_). The rhodium mass fraction in each solution was calculated via:

1 $$\left[ {Rh, ng/g} \right] = \frac{{26.55*\left( {m_{6} - m_{5} } \right)}}{{m_{7} - m_{4} }}$$

After the solution was measured by the ICP-MS, the mass fraction of each element in the tile sample was calculated by:

2 $$\left[ {M, \mu g/g} \right]_{SemiQuant} = \frac{{\left[ {Rh} \right]*\frac{{M_{CPS} }}{{Rh_{CPS} }}*\left( {m_{7} - m_{4} } \right)*\frac{{m_{3} - m_{2} }}{{m_{5} - m_{4} }} \div 1000}}{{m_{1} }}$$

where *M*_*CPS*_ and *Rh*_*CPS*_ are the respective ICP-MS intensities of the targeted element and Rh in counts per second (CPS).

For quality assurance, duplicate samples of SRM 2859 Restricted Elements in Polyvinyl Chloride were processed similarly and measured alongside the TCR samples. The measured results were compared to the certified values, and the SemiQuant survey measurements were validated by the recoveries of 95% for chromium, 85% for lead, 83% for cadmium, and 80% for copper.

### FullQuant measurements of metals in the samples

Guided by the SemiQuant survey results, a standard mixture at an appropriate mass fraction for each of the selected 10 elements (Be, Cr, Cu, As, Se, Cd, Sb, Ba, Tl, Pb) was made from NIST 3100 series SRMs (see SI). About 1 mL of the digested sample solution was transferred to a 15 mL Falcon tube (m_8_) and weighed (m_9_). The solution was diluted with 2% HNO_3_ (v/v) to about 10 mL and weighed (m_10_). For samples selected for recovery studies, a spike of the standard mixture was added before the contents were diluted to 10 mL and weighed. Each microwave digest described above was measured with the external calibration standards prepared from NIST 3100 series SRMs for the quantitative determination of these 10 elements, [M, ng/g]_Cal_. The final mass fraction of the metals in Samples S1–S6 were calculated by:

3 $$\left[ {M, \mu g/g} \right]_{FullQuant} = \frac{{\left[ {M,ng/g} \right]_{Cal} *\left( {m_{10} - m_{8} } \right)*\frac{{m_{3} - m_{2} }}{{m_{9} - m_{8} }} \div 1000}}{{m_{1} }}$$

The detection limits were Be (0.006 μg/g), Cr (0.025 μg/g), Cu (0.20 μg/g), As (0.31 μg/g), Se (1.8 μg/g), Cd (0.023 μg/g), Sb (0.015 μg/g), Ba (0.054 μg/g), Tl (0.014 μg/g), and Pb (0.044 μg/g). An indium internal standard was introduced by a separate flow path into the ICP torch for each sample and the relative standard deviation of signal at 115 u was 4.3% throughout the measurement, which verified the stability of the instrument. One of the replicates from each Sample S2 and Sample S5 was spiked with three times the SemiQuant measured mass fraction. Except for Se which was below the detection limit, acceptable recoveries in the range of 71–103% were achieved for the selected elements according to EPA Method 200.8^38^.

### Method of wiping for measuring the surface concentration of metals

A method of composite wiping for Pb content on common household surfaces (e.g., floors or window sills) developed by EPA^31^ was followed. The Whatman 542 hardened ashless filter papers were utilized as surface wipes to simulate a child’s hand touching the playground flooring surface. The filter paper was cut into quarters and wetted with 25 μL sub-boiling distilled water. Each quarter was used to wipe the same sample surface and the surface area was measured (*S*, *ft*^*2*^). All four wipes used to wipe a single sample were combined in a 15 mL Falcon tube for the next steps of sample preparation. Based on the approach developed by EPA^31^ for picking up Pb on the surface, this procedure was sufficient for picking up the surface metal elements by simulating children touching the TCR playgrounds.

The wipes, all four quarters together, were placed in the PTFE vessels of a Multiwave GO Plus microwave digestion system (Anton Paar, Ashland, VA, USA). A 2.0 mL aliquot of (67–70) % mass fraction HNO_3_ (Sigma Aldrich, OmniTrace ICP-MS grade) and 4.0 mL deionized (DI) water were added to each vessel. EPA Method 3015A^39^ was used for the microwave digestion of the wipes with a temperature ramp to 180 °C within approximately 10 min followed by a hold for another 5 min. The digested solution was transferred into a pre-weighed 50 mL Falcon tube (m_11_) and diluted with DI water to 50 mL followed by weighing (m_12_). All the samples were centrifuged to separate the precipitates^40^ and the top layer of supernatants was transferred to a 15 mL Falcon tube for measurement by ICP-MS.

The microwave digestion of the wipes was validated by spiking the filter wipes directly with an SRM standard mixture of the selected 10 elements. Three replicates were carried out and the recoveries for each element are shown in Table S2 to be within 82–105%, which validated the method.

### Accelerated outdoor weathering by NIST SPHERE (simulated photodegradation via high energy radiant exposure)

NIST’s SPHERE can illuminate samples with a high level of UV irradiance of up to 60 or more suns and provides the control of temperature and relative humidity as weathering parameters^32,37^. The SPHERE has been successfully applied to accelerate the weathering of polymers^33,34^ and nanocomposites^35,36^. Although a direct comparison to outdoor weathering cannot be made due to the complexity of weather patterns, such as cloud cover and precipitation, the total annual energy at each wavelength can be simulated using the gathered spectroradiometric data collected by SR-18 radiometers located in Miami, southern Florida. It agrees with USDA spectral power distribution (SPD) data and NREL SMARTS 2.9.5 (Simple Model of the Atmospheric Radiative Transfer of Sunshine. https://www.nrel.gov/grid/solar-resource/smarts.html) for southern Florida location^40^. The SPD of SPHERE irradiation is comparable to ASTM G-177 sunlight derived from SMARTS 2.9.5^33^. The samples of this work were exposed to UV radiation of 12.1 MJ/m^2^/day in SHPERE. Given the outdoor sunlight in southern Florida is about 280 MJ/m^2^ per year^36^, 23.2-day of SPHERE time equals 1 year’s worth of weathering by sunlight in southern Florida. Furthermore, many polymer materials, such as polycarbonate (PC) and poly(styrene-co-acrylonitrile) (CAN), have been studied by SPHERE and were found to obey reciprocity up to 400 MJ/m^2^ of SPHERE exposure^33,34^. The reciprocity was assumed to be obeyed with the increase of UV irradiation on the photodegradation of TCR in this study.

Sample S2 (green) and Sample S6 (black) were chosen for SPHERE weathering to represent different colors and different tile products. Two replicates were prepared for SPHERE weathering at 75% RH and one replicate at 0% RH. Each replicate was cut into a quarter circular shape with a surface area of *S* = 0.0075 ft^2^. For each RH, two Teflon quarter circular plates of the same size were used as blanks. Each replicate or blank was fully wiped four times by the aforementioned wiping method and combined before SPHERE weathering. The wipes were collected in a 15 mL Falcon tube and capped. After the initial surface wipings, Samples S2 and S6 tiles were placed in the SPHERE for accelerated weathering treatments^32^. A 52-day duration of exposure to high-energy UV radiation was carried out to simulate a 2.17-year weathering in southern Florida^36,41^. Only the effects of UV radiation from sunlight and moisture at ≈ 0% RH (one replicate) dry condition or ≈ 75% RH (two replicates) wet condition were simulated at a constant temperature of 55 °C. Other conditions, such as ozone oxidation, raining effect on surface metals, were not simulated. The tire crumb rubber tile samples were taken out of the SPHERE five times for composite wiping at 322 h, 579 h, 868 h, 1135 h, and 1200 h of SPHERE time, corresponding to 0.58 y, 1.05 y, 1.57 y, 2.06 y and 2.17 y of weathering outside the SPHERE, respectively.

The wipes for each replicate of samples and blanks were microwave digested as described above, and the mass fraction of the selected 10 elements, [M, ng/g]_wipes_, were determined by ICP-MS with an external calibration standard mixture prepared from NIST 3100 series SRMs. The corresponding blank-corrected MSR was calculated by:

4 $$\left[ {M, \mu g/ft^{2} } \right]_{ELT} = \frac{{\left( {\left[ {M,ng/g} \right]_{wipes} - \left[ {\overline{M},ng/g} \right]_{blank} } \right)*\left( {m_{12} - m_{11} } \right) \div 1000}}{{S, ft^{2} }}$$

## Results and discussion

### Assessment of bulk metal contents

Six samples (S1–S6) were investigated, and they are commercially available tire crumb rubber (TCR) playground tiles (see the Table in "Methods and Materials") obtained from two different U.S manufacturers that are suppliers to two major hardware stores. To select representative TCR playground tiles for metal surface release (MSR) study, a survey measurement of all six TCR samples by the ICP-MS in SemiQuant mode was firstly carried out to assess the elemental content in the samples. Figures S1–S6 show the mass fraction of elements found in the tile samples. Many metal and non-metal elements were detected from 1 ng/g to 30 mg/g in these samples. Because of the expected intrinsic inhomogeneity of rubber samples, the medians were better representative values of the general metal contents in the six TCR samples studied, as shown in Figures S1-S6. The measured metal contents were the highest for Zn (19 mg/g) and Fe (1.3 mg/g), and fell in the following ranges for other metals: 100 μg/g to 1000 μg/g for Na, Mg, Al, K, Co; 10 μg/g to 100 μg/g for Cu, Ti and Pb; 1 μg/g to 10 μg/g for Cr, Mn, Ni, As, Sn, and Ba; 0.1 μg/g to 1 μg/g for Se, Mo, Cd, In, Sb and Tl; and < 0.1 μg/g for Be and rare earth elements. No Ru, Te, Re, Os, Ir, Pt, or Au was detected. Notably, these measured values agree with those reported in the literature^1,28^.

Among the six studied samples, S4 (represented by the dark green solid circles in the figures) appears to come from a very different used-tire source because the metal contents therein were either the highest or lowest of all tile samples. The minimum amounts of sulfur (S) and zinc (Zn) measured in this sample (Figures S1 and S2) may indicate a lack of sulfur vulcanization process because ZnO is widely used as the catalysis of sulfur vulcanization in rubber manufacturing. The metal contents measured in the remaining samples showed good agreement as the differences among them were within 1 order of magnitude.

**Figure 1:**
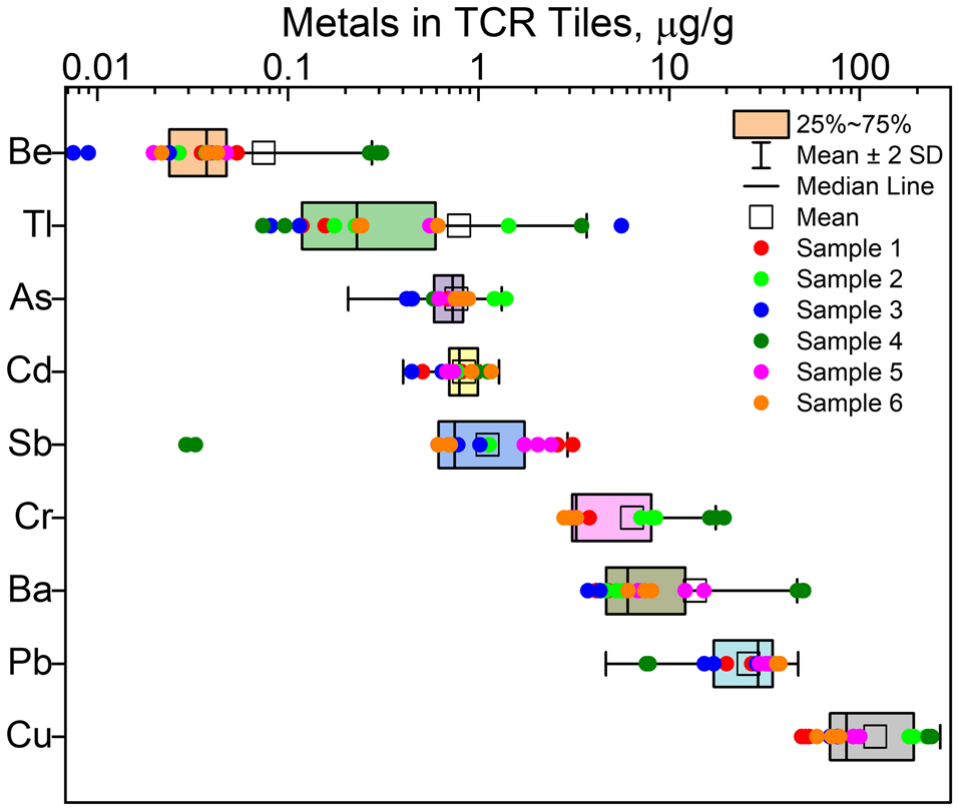
FullQuant values of metals (Be, Tl, As, Cd, Sb, Cr, Ba, Pb, Cu) in the six samples of TCR playground tiles (color-coded) with three replicates (represented by the solid circles of the same color). The bars show 25% to 75% percentile range, vertical lines indicate the median, and open squares represent the mean value for their respective metal contents. Se was below the 1.8 μg/g detection limit.

The FullQuant measurements of 10 selected elements was then carried out^30^. These elements are the most concerning for potentially causing harm to children’s health if released from the TCR tiles' surface when children play on the playgrounds. Excepting Se, which was below the 1.8 μg/g detection limit, the ranges of measured metal contents were within 10–100 μg/g for Cu and Pb; 1–10 μg/g for Ba and Cr; 0.1–1 μg/g for Sb, Cd, As, and Tl; and 0.01–0.1 μg/g for Be as shown in Fig. 1. As revealed in Figure S7, the slope and intercept of the linear correlation (*R*^*2*^ = 0.985) between SemiQuant and FullQuant for the metal contents among all the samples (S1 to S6) were close to 1 (0.976) and 0 (− 0.0423), respectively^42^, which implies that SemiQuant and FullQuant well agree with each other.

### Assessment of the surface release of 10 selected metals

**Figure 2:**
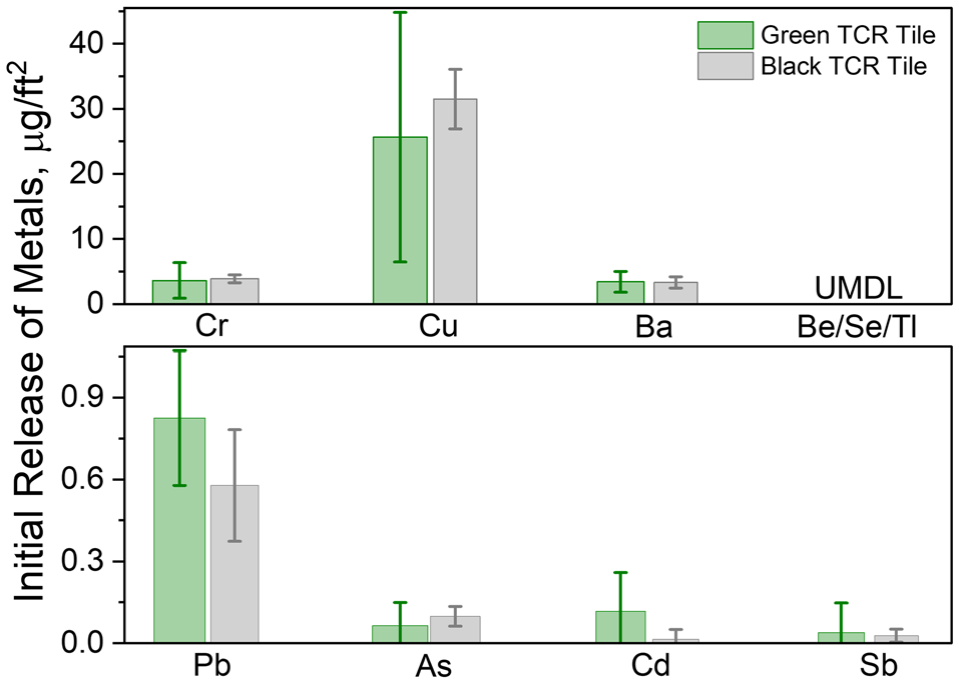
The initial surface releases (ISR) of the 10 metals on Samples S2 (green bar) and S6 (gray bar). Three replicates were performed for each and standard deviations are shown as vertical lines on the bars. Be, Se, and Tl were undetectable (UD). The unit for ISR, μg/ft^2^ is used because it is used in the regulatory standard. Use the relation of 1 μg/ft^2^ = 11 μg/m^2^ to convert to SI unit.

Samples S2 and S6 were chosen for the accelerated weathering study for their relatively higher overall contents of the 10 selected metals. To estimate the amount of harmful metals available for pickup through contact on a playground floored with tire crumb rubber (TCR) tiles, a method of composite surface wiping that simulated children’s physical contact with the floor was adopted from the EPA method used in evaluating lead (Pb) content on floors, painted walls, window sills, or other common household items^31^ (see Methods). The initial surface releases (ISR) of the 10 selected metals are plotted in Fig. 2. Except for Pb, the measured ISRs correlated positively with the corresponding bulk contents, i.e., the higher the bulk content was, the higher the surface release (Fig. 1). Ranges of ISRs were: 10–100 μg/ft^2^ for Cu, 1–10 μg/ft^2^ for Ba and Cr, 0.5–1 μg/ft^2^ for Pb, 0.01–0.5 μg/ft^2^ for As, Cd, and Sb, and below the detection limit for Be, Se, and Tl (i.e., less than the values obtained from the procedural blanks).

The μg/ft^2^ unit (1 μg/ft^2^ = 11 μg/m^2^) is used here to facilitate the comparison with values in the literature because the regulatory standard for the surface content metal is issued in this unit to be discussed below. Pb had a bulk content approximately 3 times higher, but a surface release approximately 4 times lower than Ba and Cr. Color additives of the tiles do not appear to be a determining factor for the ISRs since the green tiles showed a similar trend as the black ones.

### Cumulative release of 10 metals on TCR surface under weathering with heavy UV radiation

**Figure 3:**
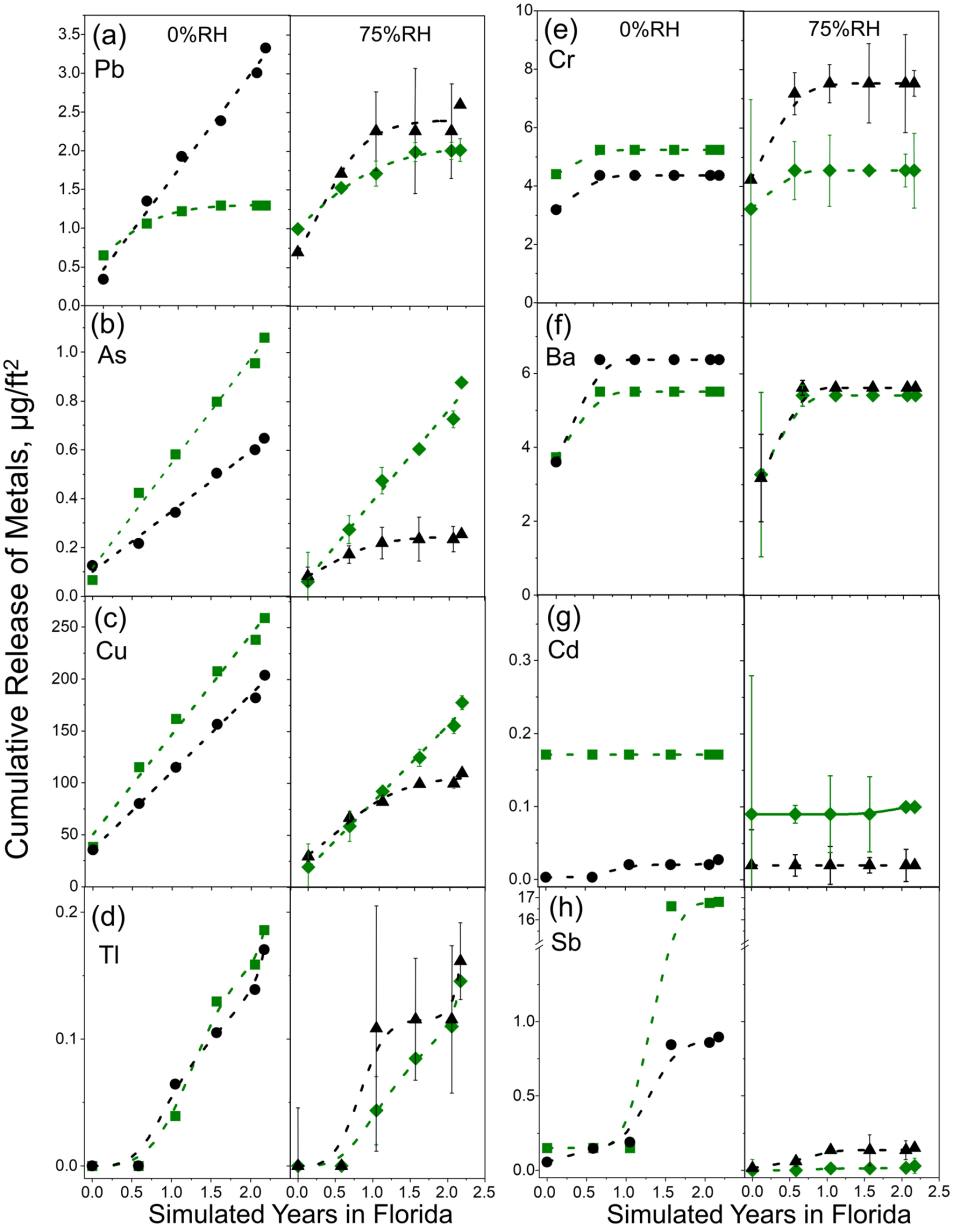
Cumulative metal surface release (MSR) of Pb (a), As (b), Cu (c), Tl (d), Cr (e), Ba (f), Cd (g) and Sb (h) as a function of accelerated weathering time achieved by NIST SPHERE on the green (Sample S2, green squares and diamonds) and black (Sample S6, black circles and triangles) TCR playground tiles. Duplicate samples were weathered at 75% relative humidity (RH, wet conditions) and one at 0% RH (dry conditions). The uncertainty was stated by one standard deviation of replicates.

The results of the cumulative metal surface releases, calculated by adding all previously measured metal surface release (MSR) values up to the point of interest, were plotted versus the simulated weathering time up to more than 2 years in Fig. 3 for Pb (a), As (b), Cu (c), Tl (d), Cr (e), Ba (f), Cd (g) and Sb (h). Be and Se were not shown because all values for the two elements were below the values of the procedural blanks obtained from the compositing wipings of the Teflon tiles placed alongside the samples in the SPHERE.

As displayed in Fig. 3, the release of bulk metals to the surface during weathering could be continuous as for Pb, As, Cu, and Tl, or stepwise as in the case of Cr, Ba, and Sb, or no release as the case for Cd. Six different MSR patterns can be identified in general, which shows the complexity of the processes that make estimating weathering-dependent surface release of metals a challenging task in general. The first is the continuous release (CR) pattern in which metal is released at a constant rate as the weathering continues. Pb (a), As (b), and Cu (c) followed this pattern under the 0% RH condition, except for Pb in Sample S2 (green tile), which followed the second growth-and-level-off release (GLR) pattern. Under the wet, i.e., 75% RH, condition, the releases of As and Cu in Sample S2 (green tile) remained in the CR pattern while Pb was released in a different GLR pattern. In contrast, the release patterns of the three metals in Sample S6 (black tile) changed from the CR under the 0% RH to the GLR under the 75% RH condition, which demonstrates the marked effect of humidity on the black tiles and suggests that the wet condition could suppress the release of these three metals in Sample S6. The markedly different effect of humidity on the green vs. black TCR tiles during weathering might have resulted from the different material properties caused by the coloring process for manufacturing the TCR tiles.

Similar CR patterns were observed for As and Cu (Fig. 3b,c, RH = 0%) though the rate of release differed for green and black tiles, which were at (0.43 ± 0.02) μg/ft^2^/y (*p* < 0.001) and (0.25 ± 0.01) μg/ft^2^/y (*p* < 0.001) for As, and (96 ± 6) μg/ft^2^/y (*p* < 0.001) and (74 ± 3) μg/ft^2^/y (*p* < 0.001) for Cu, respectively. The results of As and Cu showed that after a year of weathering, the amount of MSR is greater albeit on the same order as ISR (Fig. 2 and Table S1), indicating the significance of weathering on the release of the two elements.

The third is the inductive CR (ICR) pattern whereby CR starts after an initial induction period with no surface release. The release of Tl adhered to this pattern under the 0% RH condition (Fig. 3d) with a half-year induction period followed by CRs at approximately the same rate of ≈ (0.10 ± 0.01) μg/ft^2^/y (*p* = 0.0014) for both Sample S2 and Sample S6, which is consistent with that both samples had the same bulk Tl contents at (0.22 ± 0.01) μg/g as measured by the SemiQuant method (Table S1). A similar ICR pattern was also observed for Tl under the 75% RH condition with the release rate at ≈(0.10 ± 0.03) μg/ft^2^/y (*p* = 0.005 to 0.006) after the half-year point.

The fourth is the step release (SR) pattern that was followed by Cr (Fig. 3e) and Ba (Fig. 3f) in which only one small step increase beyond the ISR was observed after a half-year weathering. The amounts of increase were about 30–80% of their ISR values and no marked humility effect was observed.

The fifth is the no-weathering-release (NR) pattern that was represented by Cd (Fig. 3g) in which weathering did not cause any further metal release beyond the ISR. Considering the measurement uncertainties (vertical lines), no obvious humidity effect was observed. In this case, the ISR would be the value for assessing the exposures.

The sixth and last MSR pattern is the singular release (SiR) pattern as followed by Sb (Fig. 3h). The ISRs of Sb in Sample S2 and Sample S6, (0.09 ± 0.08) μg/ft^2^ and (0.03 ± 0.02) μg/ft^2^ respectively, were relatively small. However, the singular large releases at 17 μg/ft^2^ and 0.84 μg/ft^2^ from Sample S2 and Sample S6 respectively, were observed at the 1.57-year of weathering mark under the 0% RH condition. No significant cumulative releases were observed thereafter. The reason could be that such SiR could be a random surface release of aggregated metal particles caught by the wipings, which could happen at any time due to its random nature as the TCR aged under the weathering condition.

Although it would be difficult to study the SiR systematically, the relative standard deviation (RSD) of measuring the bulk metal content could be an informative parameter that would help predict the eventual occurrence of SiR. For instance, in the case of Sb here, the three replicates of the SemiQuant/FullQuant measurements of Sample S2 with a sample size of 0.25 g gave the bulk metal contents of (0.74, 1.07, 1.11) μg/g and (0.72, 1.14, 1.13) μg/g, respectively. These measured replications show a relatively larger RSD of > 20%, compared to Pb (≈5%), As (≈9%), Cu (≈12%), Tl (≈6%), Cr (≈10%), Ba (≈10%), indicating a highly inhomogeneous distribution of Sb in Sample S2 that could be caused by the existence of aggregated particles of high metal content. Applying the same reasoning to Cd, which also has a similarly large RSD of 24%, one would expect that the SiR could take place given sufficient time even though it was not detected in the period shown in Fig. 3g.

### Discussion on the potential exposure, mitigation, and policy implications

To the best of our knowledge, Pb is the only element to have environmental safety regulatory standards for bulk and surface contents. Under the Toxic Substances Control Act (TSCA, US EPA), a limit of 400 μg/g in bare soil is the safety standard for Pb bulk content for children's play areas, and 10 μg/ft^2^ (40 μg/ft^2^ before 2020) is the safety standard for Pb surface content in residential areas. Since Pb is the only element that has a regulatory limit for its surface contents, the potentially harmful surface content levels for other elements, as the inferred limits, were estimated from the limits for their bulk content using a surface-to-bulk content ratio (StB, vide infra) of 0.025 derived from EPA regulations for Pb on the surface and in the bulk.

Collected in Table 1 are the regulatory or advisory limits for the bulk contents of the 10 selected metals from diverse authoritative sources. The bulk content standards for Sb, As, Ba, Cd, Cr, and Se are from the maximum allowable soluble concentrations listed in ASTM F3012-14 for loose-fill rubber used for playgrounds^43^. Those for Be and Tl are from the maximum permissible concentrations of metals in soil published by the Dutch National Institute for Public Health and the Environment (RIVM 601,501,001)^44^. The Cu standard is adopted from the US EPA biosolid standard (40 CFR Part 503)^45^. Using the StB ratio of 0.025 for Pb based on current EPA rules, the bulk content standards for other toxic elements were converted to surface content equivalent as listed in Table 1. Employing the MSR data shown in Fig. 3, the potential release of toxic metals within 3 years of weathering was compared to the aforementioned limits for removable surface contents. Cr was the only element with its mean ISR at (3.62 ± 2.73) μg/ft^2^ (Sample S2) and (3.87 ± 0.61) μg/ft^2^ (Sample S6) exceeding the inferred safety limit of 1.55 μg/ft^2^. The MSRs of Be, Se, Cd, Pb, and Ba would not exceed their inferred limits within 3 years. However, the MSR of Cu would reach its inferred limit at ca. 0.7 y for S2 and ca. 1 y for S6. It would take between 1.5 y and 2.5 y for the MSR of As in Sample S2 to reach the inferred limit under either the dry or wet condition and in Sample S6 under the dry condition. For Tl, the safety limit could be reached at about 1 y in both Sample S2 and Sample S6 under either dry or wet conditions. Finally, the MSR of Sb could be far larger than the inferred safety limit if the SiR takes place.

**Table 1:** The release models of 10 selected metals and the potential exposure to children.

Metals	Standards, μg/g	Inferred limits, μg/ft^2^	Humidity effect	Models	Harm on confirmation or prediction	Time to harm within 3 year
Be	1.1(RIVM)	0.028	na	na	No	No Harm
Cr	61.9(ASTM)	1.55	No	SR	Yes on C	0
Cu	4300(EPA)	107	Yes*	CR(0H),GLR(75H)	Yes on C	≈1
			or No	CR	Yes on C	≈0.7
As	25.8(ASTM)	0.64	Yes*	CR(0H),GLR(75H)	No(75H), Yes on P(0H)	≈2.5
			or No	CR	Yes on C	≈1.5
Se	515.8(ASTM)	12.9	na	na	No	No Harm
Cd	77.4(ASTM)	1.93	No	NR	No	No Harm
Sb	61.9(ASTM)	1.55	No	SiR	No or Yes on C	Any(one time)
Ba	1031(ASTM)	25.8	No	SR	No	No Harm
Tl	1.3(RIVM)	0.033	No	ICR	Yes on C	≈1
Pb	400(EPA)	10(EPA)	Yes*	CR(0H),GLR(75H)	No	No Harm
			or No	GLR	No	No Harm

*SR:* Step Release, *CR:* Continuous Release, *ICR:* Inductive Continuous Release, *GLR:* Grow and Level off Release, *NR: No* Accumulated Release, *SiR:* Singular Release, *75H:* 75% Humidity, *0H: 0*% Humidity, na: not applicable.

*only for black tile.

The weathering conditions in the SPHERE did not consider the cleansing effect of precipitation, such as rain. Assuming that the cleansing effect of precipitation is equivalent to wipings in the experiment, the amount of metal available for human exposure is determined by the rate of the metal’s release on the TCR tile surface and the frequency of rainfalls. Assuming a constant rate of release between the neighboring two points in each plot shown in Fig. 3, the rate of release is calculated as the slope defined by the neighboring two points. Table 2 lists the maximum rate of release for the ten selected metals from the green (Sample S2) and black (Sample S6) TCR tiles. Regarding the frequency of rainfalls, Tucson, Arizona is one of the driest cities in the United States, which has at least one rainy day per month^46^. Assuming a precipitation frequency of at least once a month for an average city in the US, the maximum amount of accumulated metal accessible through casual contact is calculated to be the maximum rate of release of the metal multiplied by one month. Numerically, the maximum accumulation values equal those listed in Table 2. A comparison of the maximum accumulation in Table 2 to the inferred exposure limits in Table 1 shows that all metals on the TCR tiles are at or below the inferred exposure limit except for Sb on Sample S2 (Green tile) at 0% humidity, which exceeded the inferred limit of 1.6 µg/ft^2^.

**Table 2:** Maximum rate of release of metals from TCR surface in µg/ft^2^/month*.

Sample, RH	Cr	Cu	As	Se	Cd	Sb	Ba	Tl	Pb
S2, 0%	0.12	15	0.075	0.039	0	2.6	0.25	0.019	0.059
S6, 0%	0.17	16	0.034	0.014	0.0050	0.10	0.40	0.023	0.230
S2, 75%	0.19	16	0.110	0.037	0.0017	0.010	0.31	0.026	0.076
S6, 75%	0.42	7	0.014	0.071	0	0.013	0.35	0.033	0.240

*Be is excluded because its release rate is 0.

In summary, TCR tiles have seen wide use in children’s playgrounds; yet the safety of the material is not fully assessed due to a lack of information on how much toxic metals were released from the surface of TCR tiles and a lack of regulatory standards for the maximum allowable level of toxic metals on the surface. the MSR from TCR tiles was measured and compared the results to ad hoc inferred safety limits derived from the regulatory standards for bulk and surface metal contents. Of the ten selected toxic metals of this study (Be, Cr, Cu, As, Se, Cd, Sb, Ba, Tl, Pb), Cr was the only element whose initial contents on the surface of some TCR test sample tiles exceeded the inferred limit, suggesting a rinse of the playground with water after the installation is advisable. Taking into account the cleansing effect of precipitation, only Sb of the ten select toxic elements may exceed the inferred safety limit within the first 3 years of service. It is important to point out that Pb is the only toxic metal that has an established regulatory limit for its content on the surface and the Pb found on the TCR tiles of this work is below the limit; therefore, there is no regulatory ground to consider the TCR tiles as unsafe. Consequently, the work revealed an urgent need for regulatory guidance for the surface contents of other toxic metals to further the safety study of TCR tiles, and the methodology developed herein could stimulate the endeavor.

### Supplementary Information

Supplementary Information.

## Data Availability

The data discussed in this study can be found in the Supporting Material.
