# Supplementary material for: Assessing children’s potential exposures to harmful metals in tire crumb rubber by accelerated photodegradation weathering
Source: Sci Rep. 2023 Aug 24;13:13877. doi: 10.1038/s41598-023-38574-z (PMC10449860; doi:10.1038/s41598-023-38574-z)
Supplement: Supplementary file 1 — Supplementary Information. [file 41598_2023_38574_MOESM1_ESM.pdf]

Supporting Information

Assessing Children's Potential Exposures to Harmful Metals in Tire Crumb Rubber by Accelerated Photodegradation Weathering

Robyn Winz[1,2], *Lee L. Yu*[2*], *Li-Piin Sung*[2], *YuYe J. Tong*[1], *Dejun Chen*[1*]

1. Environmental Metrology and Policy Program, Graduate School of Arts and Sciences, Georgetown University, Washington, DC 20057. 2. National Institute of Standards and Technology, Materials Measurement Laboratory, Chemical Sciences Division, Gaithersburg, MD 20899

Correspondence and requests for the materials should be addressed to L.Y. (email: lee.yu@nist.gov) or D.C. (email: dc424@georgetown.edu)

Content in Supporting Information:

Additional Method and Materials

Figures S1 to S7

Tables S1 to S2

Additional Method and Materials:

Materials and Reagents

Optima Grade nitric acid at nominal 67 % to 69 % mass fraction from Fisher Chemical (lot no. 1220080) and OmniTrace nitric acid at nominal 67 % to 70 % mass fraction from Sigma Aldrich (lot no. 60309) were used for microwave digestion of samples and ICP-MS solvent, respectively. For the semi-quantitative survey measurements of all digested samples, NIST SRM (Standard Reference Material) 3144 Rhodium (Rh) Standard Solution (lot no. 170930) was used as the internal standard, and NIST SRM 2859 Restricted Elements in Polyvinyl Chloride (ID 170814) was used as a quality control sample. For recovery studies in method development of wipes, High Purity Standards 31 components ICP-MS standard (2014936-100, Ag, Al, As, B, Ba, Be, Ca, Cd, Co, Cr, Cu, Eu, Ho, La, Li, Mg, Mn, Mo, Na, Ni, Pb, Sb, Sc, Se, Sr, Th, Tl, U, V, Yb, Zn) and an indium standard (Supelco, TraceCERT 00734) were used as calibration standards and internal standard, respectively. The following NIST SRMs were used to prepare the multi-element solution for quantitative analysis of the digested samples and the processed wipes:

1. Lead SRM 3128, lot no. 030721
2. Antimony SRM 3102a, lot no. 140911
3. Copper SRM 3114, lot no. 120618
4. Selenium SRM 3149, lot no. 100901
5. Chromium SRM 3112a, lot no. 170630
6. Arsenic SRM 3103a, lot no. 100818
7. Barium SRM 3104a, lot no 140909
8. Thallium SRM 3158, lot no. 151215
9. Cadmium SRM 1308, lot no. 130116
10. Beryllium SRM 3105a, lot no. 090514

Whatman 542 hardened ashless filter papers (55 mm, category number 1542055) were used as surface wipes to mimic children's hand contact with the playground.

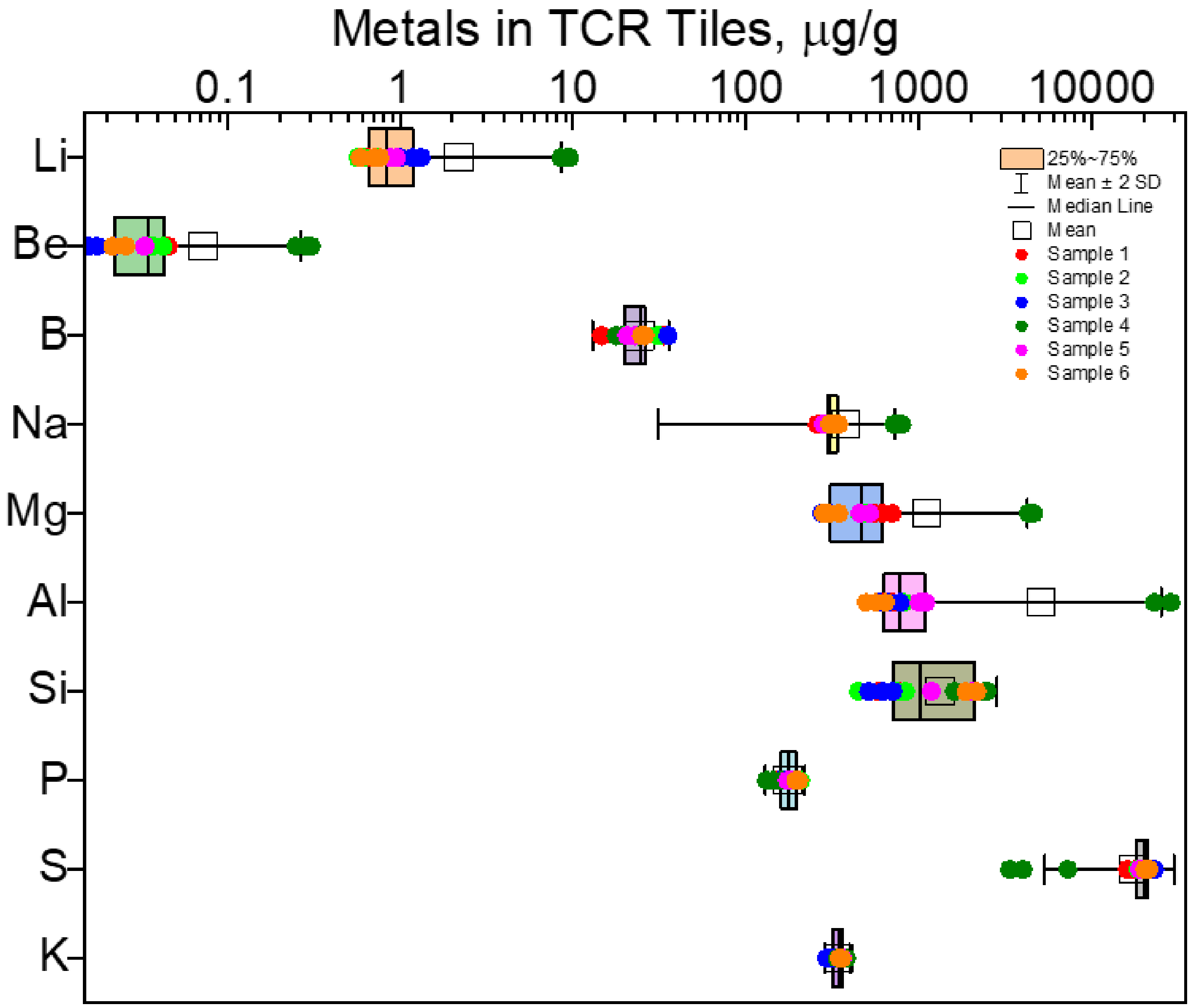

Figure S1. The bar figure of SemiQuant results of elements (Li, Be, B, Na, Mg, Al, Si, P, S, K) in the tire crumb rubber (TCR) playground tiles (μg/g) for 6 samples (color dots) with three replicates (same color dots). The bar showed the 25th percentile to 75th percentile distribution of the metal content, and the vertical line was for the median value while the open square was the mean value.

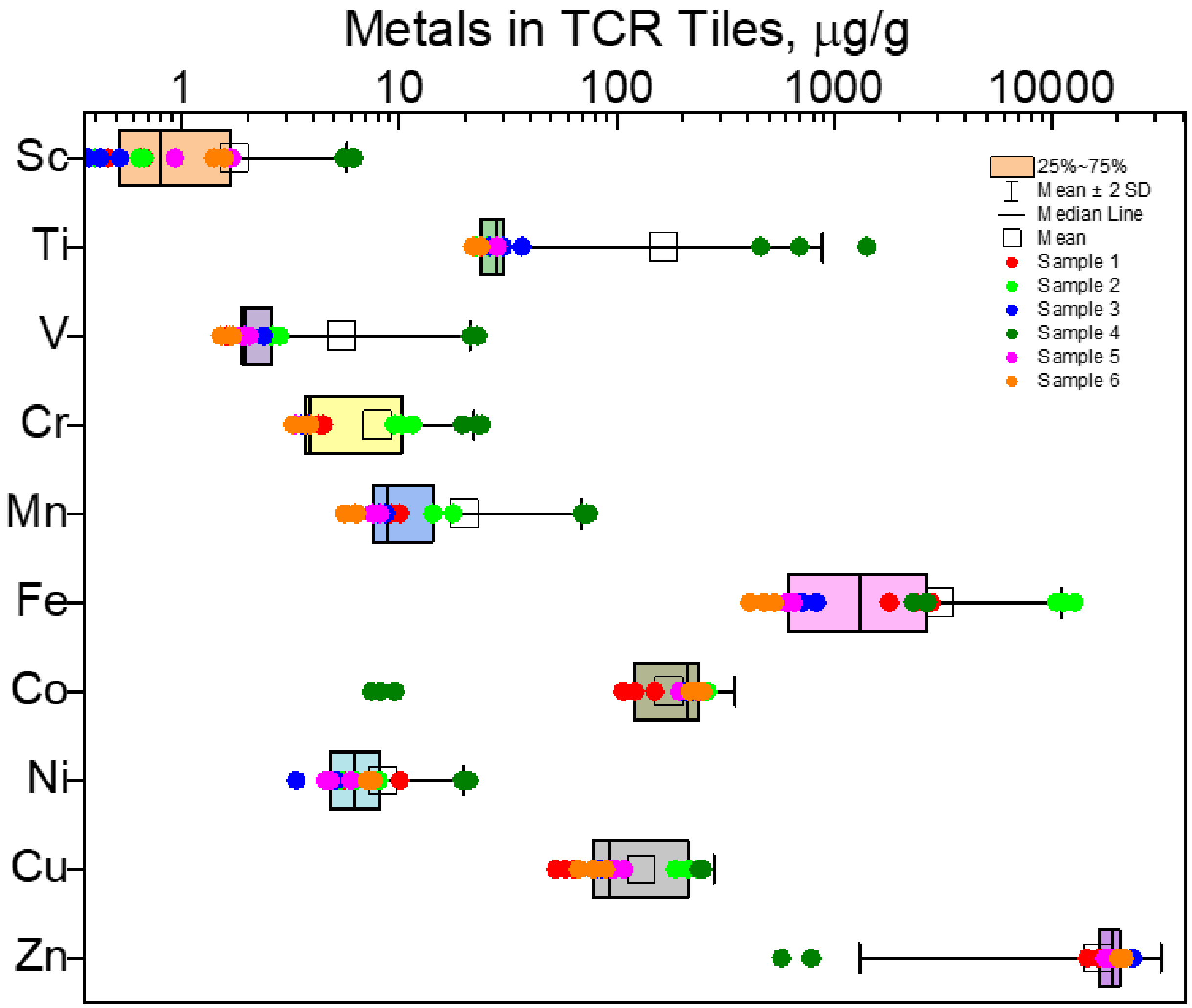

Figure S2. The bar figure of semi-quantification of elements (Sc, Ti, V, Cr, Mn, Fe, Co, Ni, Cu, Zn) in TCR recycled rubber playground tiles (µg/g) for 6 samples (color dots) with three replicates (same color dots). The bar showed the 25th percentile to 75th percentile distribution of metal content, and the vertical line was for the median value while the open square was the mean value.

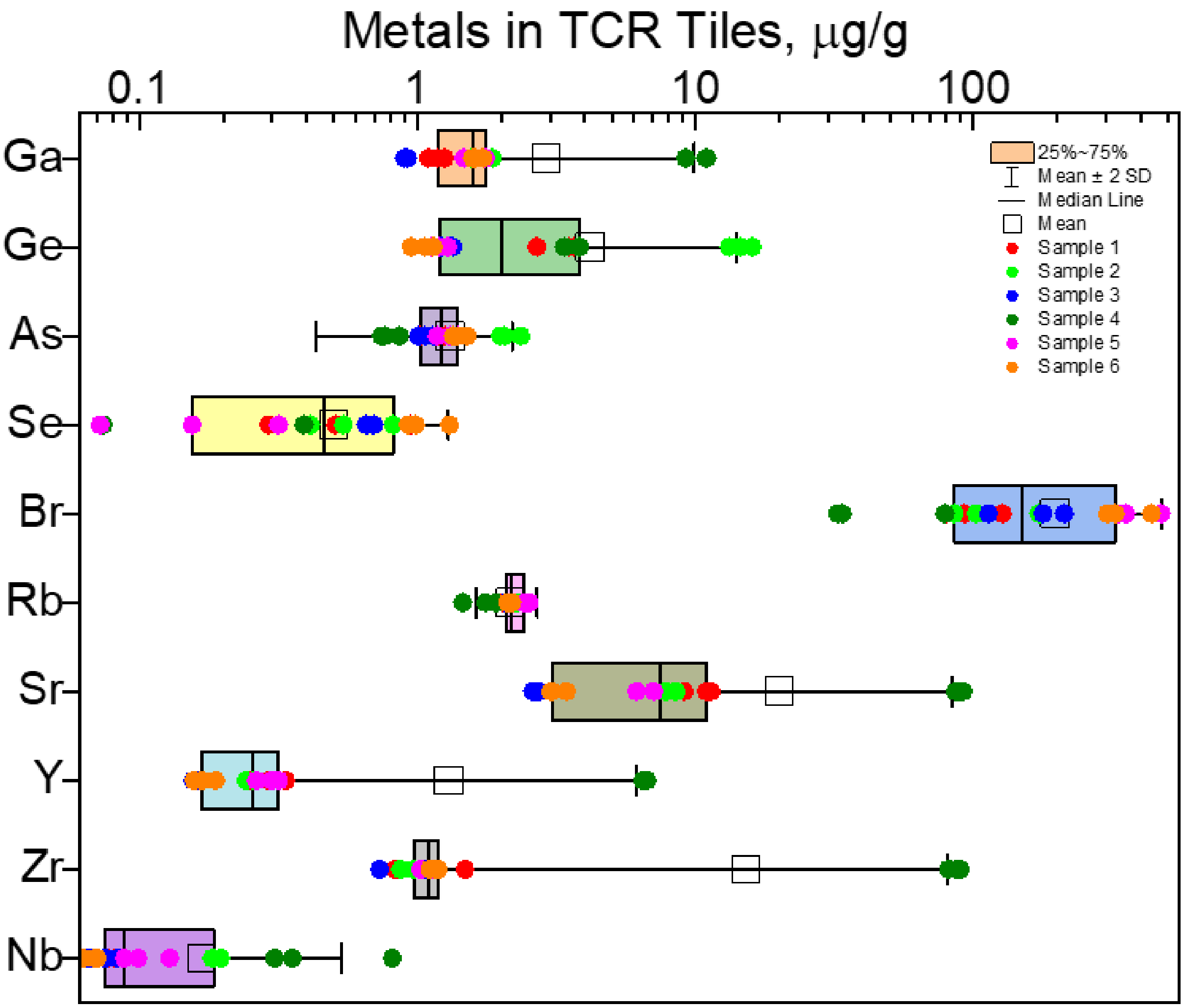

Figure S3. The bar figure of semi-quantification of elements (Ga, Ge, As, Se, Br, Rb, Sr, Y, Zr, Nb) in TCR recycled rubber playground tiles (μg/g) for 6 samples (color dots) with three replicates (same color dots). The bar showed the 25th percentile to 75th percentile distribution of metal content, and the vertical line was for the median value while the open square was the mean value.

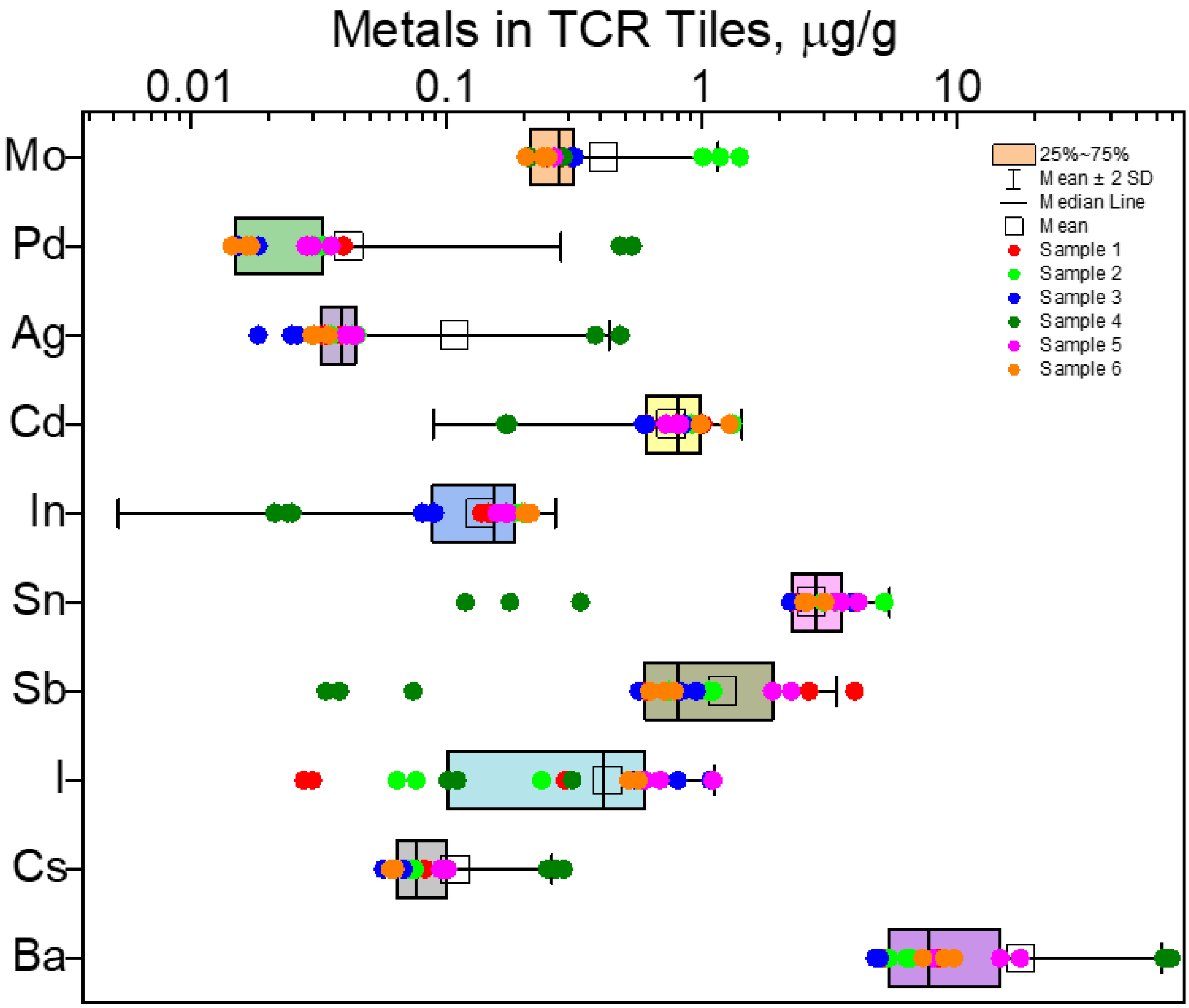

Figure S4. The bar figure of semi-quantification of elements (Mo, Pd, Ag, Cd, In, Sn, Sb, I, Cs, Ba) in TCR recycled rubber playground tiles (µg/g) for 6 samples (color dots) with three replicates (same color dots). The bar showed the 25th percentile to 75th percentile distribution of metal content, and the vertical line was for the median value while the open square was the mean value.

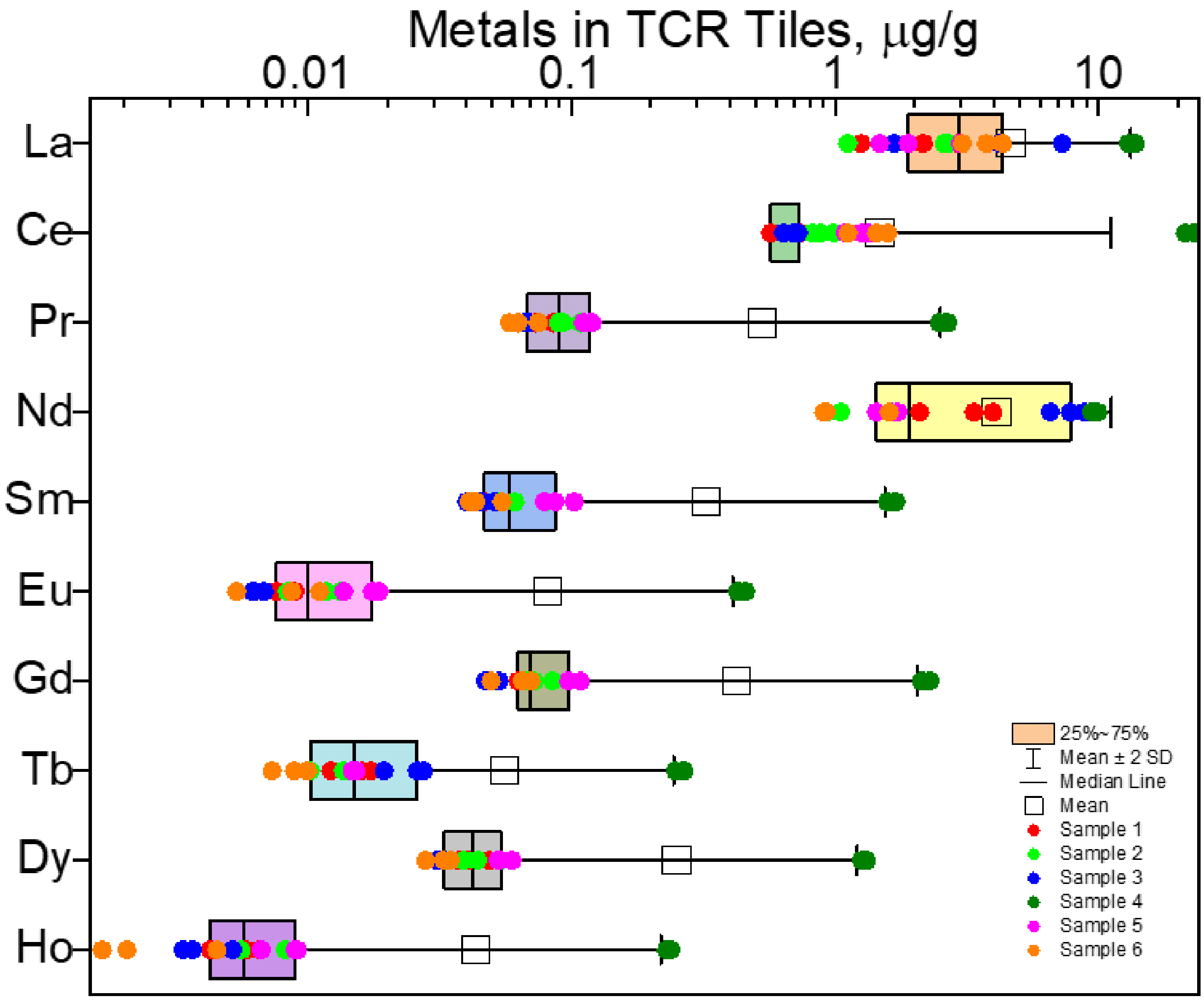

Figure S5. The bar figure of semi-quantification of elements (La, Ce, Pr, Nd, Sm, Eu, Gd, Tb, Dy, Ho) in TCR recycled rubber playground tiles (µg/g) for 6 samples (color dots) with three replicates (same color dots). The bar showed the 25th percentile to 75th percentile distribution of metal content, and the vertical line was for the median value while the open square was the mean value.

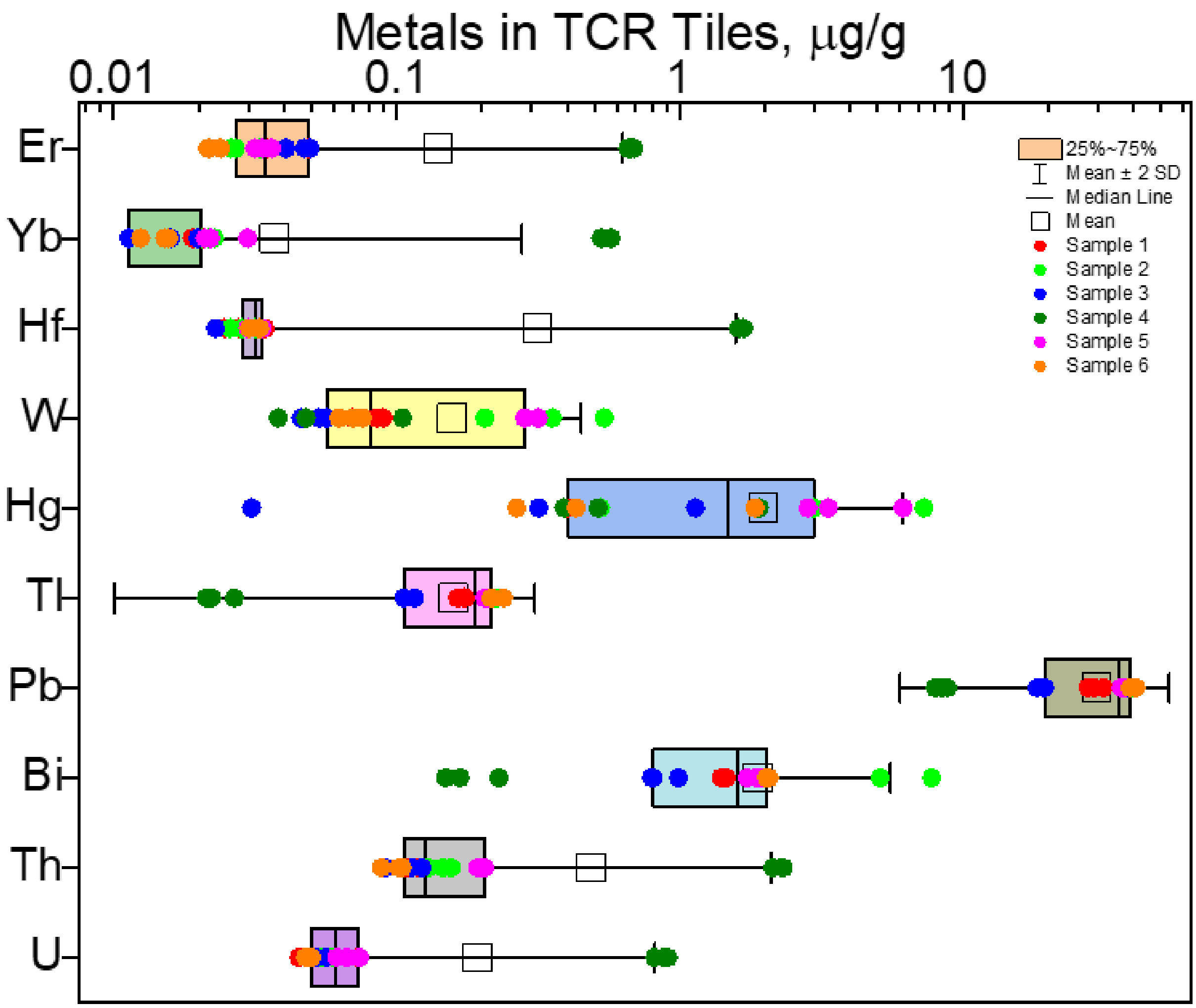

Figure S6. The bar figure of semi-quantification of elements (Er, Yb, Hf, W, Hg, Tl, Pb, Bi, Th, U) in TCR recycled rubber playground tiles (µg/g) for 6 samples (color dots) with three replicates (same color dots). The bar showed the 25th percentile to 75th percentile distribution of metal content, and the vertical line was for the median value while the open square was the mean value.

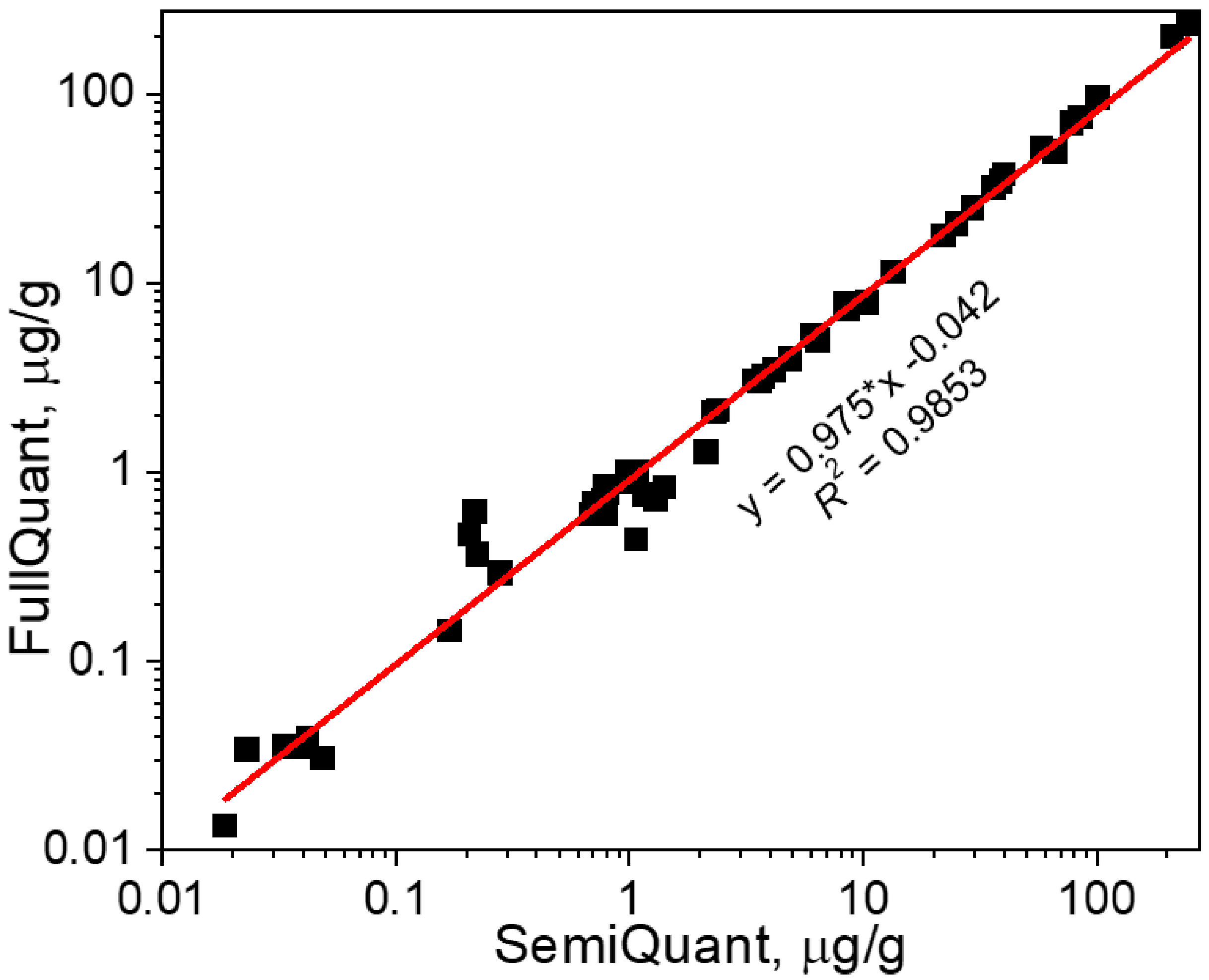

Figure S7. The correlation plot of SemiQuant and FullQuant for all samples. Each point represents one element (Be, Cr, Cu, As, Se, Cd, Sb, Ba, Tl, Pb) in a certain sample. Three outliers, one Cd and two Tl data points, were not included because of the inhomogeneity of metals in the samples.

Table S1. The SemiQuant, FullQuant, and initial surface release of metals for samples S2 and S6 used in the SPHERE experiment (the uncertainties are stated by 1 standard deviation from replicates)

Metals	SemiQuant, μg/g		FullQuant, μg/g		Surface Initial Release, μg/ft²	
	**Sample 2**	**Sample 6**	**Sample 2**	**Sample 6**	**Sample 2**	**Sample 6**
**Be**	0.038±0.004	0.023±0.002	0.035±0.007	0.034±0.011	UDL	UDL
**Cr**	10.42±1.03	3.60±0.34	7.87±0.69	3.02±0.22	3.62±2.73	3.87±0.61
**Cu**	211±24	77±11	199±21	70±9	25±19	31±4
**As**	2.13±0.20	1.41±0.08	1.26±0.10	0.82±0.06	0.11±0.06	0.10±0.04
**Se**	0.59±0.21	1.07±0.20	UDL	UDL	UDL	UDL
**Cd**	1.03±0.25	1.08±0.17	0.87±0.21	1.00±0.14	0.20±0.04	0.028±0.036
**Sb**	0.97±0.20	0.71±0.08	0.99±0.24	0.68±0.05	0.09±0.08	0.028±0.024
**Ba**	6.1±0.6	8.6±1.2	5.2±0.5	7.2±1.0	3.4±1.6	3.3±0.9
**Tl**	0.219±0.013	0.224±0.013	0.612±0.714	0.364±0.216	UDL	UDL
**Pb**	39.0±1.5	40.2±1.0	34.4±1.7	37.4±0.7	0.82±0.24	0.57±0.20

Table S2. The recovery of metals from microwave digestion of wipes

Metals	Replicate 1	Replicate 2	Replicate 3
**Be**	97.7 %	105.0 %	101.8 %
**Cr**	99.4 %	89.5 %	88.6 %
**Cu**	94.6 %	96.7 %	96.0 %
**As**	98.7 %	101.0 %	101.3 %
**Se**	92.4 %	89.2 %	92.1 %
**Cd**	92.0 %	94.2 %	92.9 %
**Sb**	84.1 %	85.9 %	85.4 %
**Ba**	87.0 %	89.0 %	87.2 %
**Tl**	83.6 %	82.9 %	82.4 %
**Pb**	88.1 %	89.5 %	87.5 %
